# Supplementary material for: OsMYB58 Negatively Regulates Plant Growth and Development by Regulating Phosphate Homeostasis
Source: Int J Mol Sci. 2024 Feb 12;25(4):2209. doi: 10.3390/ijms25042209 (PMC10889527; doi:10.3390/ijms25042209)
Supplement: Supplementary file 1 [file ijms-25-02209-s001.zip › ijms-2756057-supplementary.pdf]

Supporting information

OsMYB58 negatively regulates plant growth and development by regulating phosphate homeostasis

Dongwon Baek[1,†], Soyeon Hong [2,†], Hye Jeong Kim [3], Sunok Moon [4], Ki Hong Jung [4], Won Tae Yang [3,*], and Doh Hoon Kim [3,*]

[1] Plant Molecular Biology and Biotechnology Research Center, Gyeongsang National University, Jinju 52828, Republic of Korea
[2] National Agrobiodiversity Center, National Institute of Agricultural Sciences, Rural Development Administration, Jeonju 54874 , Republic of Korea
[3] College of Life Science and Natural Resources, Dong-A University, Busan 49315, Republic of Korea
[4] Graduate School of Biotechnology and Crop Biotech Institute, Kyung Hee University, Yongin 17104, Republic of Korea

[†] These authors contributed equally to this work
[*] Correspondence: wtyang@dau.ac.kr Tel.: +82-51-200-7507 (W.T.Y.), dhkim@dau.ac.kr; Tel.: +82-51-200-7507 (D.H.K.)

Supplementary Materials:
Supplementary Figure S1. Transcriptional expression of *OsMYB58* in rice under nutrient deficiency conditions.
Supplementary Figure S2. Transcriptional expression of *OsMYB63* in rice under nutrient deficiency conditions.
Supplementary Table S1. Lists of primers in this study
Supplementary Table S2. The shoot and root biomass of Arabidopsis wild-type Col-0 and *OsMYB58*-AraOX plants
Supplementary Table S3. The shoot and root biomass of rice wild-type, *OsMYB58*-OX, and *OsMYB58*-KO plants

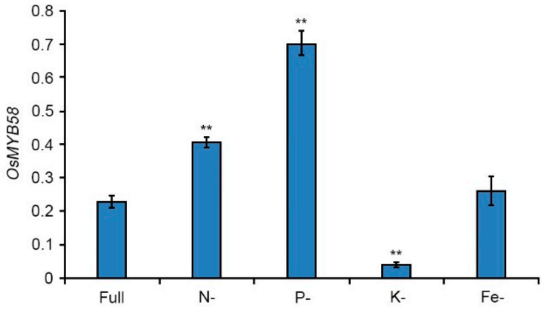

Supplementary Figure S1. Transcriptional expression of *OsMYB58* in rice under nutrient deficiency conditions. For qRT-PCR analysis, total RNA extracted from 7-day-old seedlings of rice wild-type plants (*Oryza sativa* L. 'Ilmi') growing in various nutrient deficiency conditions. Rice plants were transferred to nitrogen (N-; 0.25 mM), phosphate (P-; 0.0125 mM), potassium (K-; 0.01 mM), or iron (Fe-; 0.01 mM)-deficient media and grown for 7 days. The qRT-PCR analyzed the transcript levels of the *OsMYB58* gene using specific primers in Supplementary Table S1. Expression of *OsACTIN1* was used for the normalization. Error bars represent the mean ± standard deviation (SD) of three biological replicates. Asterisks represent significant differences from the full sample (**; p-value ≤ 0.01, Student's t-test).

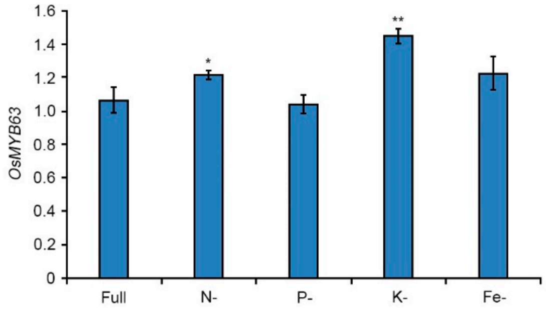

Supplementary Figure S2. Transcriptional expression of *OsMYB63* in rice under nutrient deficiency conditions. For qRT-PCR analysis, total RNA extracted from 7-day-old seedlings of rice wild-type plants (*Oryza sativa* L. 'Ilmi') growing in various nutrient deficiency conditions. Rice plants were transferred to nitrogen (N-; 0.25 mM), phosphate (P-; 0.0125 mM), potassium (K-; 0.01 mM), or iron (Fe-; 0.01 mM)-deficient media and grown for 7 days. The qRT-PCR analyzed the transcript levels of the *OsMYB63* gene using specific primers in Supplementary Table S1. Expression of *OsACTIN1* was used for the normalization. Error bars represent the mean ± standard deviation (SD) of three biological replicates. Asterisks represent significant differences from the full sample (*; 0.01 < p-value ≤ 0.05; **; p-value ≤ 0.01, Student's t-test).

Supplementary Table S1. Lists of primers in this study

Gene	Direction	Sequence (5′→3′)	Purpose
OsMYB58-RT	Forward	CATCGCCTACATCCAGAAG	Analysis of qRT-PCR or RT-PCR
	Reverse	GAGATGTCGATGTCTTGCTC	
OsACTIN-RT	Forward	ATGCTCTCCCCCATGCTATC	
	Reverse	TCTTCCTTGCTCATCCTGTC	
AtTUBULIN2-RT	Forward	TGGCATCAACTTTCATTGGA	
	Reverse	ATGTTGCTCTCCGCTTCTGT	
OsmiR399a-qRT	Forward	GCTGGAAATGATGCTGGTAGC	
	Reverse	CTCCTTTGGCACGAGATCTGT	
OsmiR399j-qRT	Forward	GGAGCATGTAAGTCTTTTGTAGC	
	Reverse	GGCAACTCTCCTTTGGCAGA	
OsIPS1-qRT	Forward	CTAAGGTAGGGCAACTTGTATC	
	Reverse	TTATTAGAGCAAGGACCGAAAC	
OsPHO2-qRT	Forward	GGTGCAGCTGGAACACCTTA	
	Reverse	GCACCGGAATGGTAGTGAA	
OsPT2-qRT	Forward	GACGAGACCGCCCAAGAAC	
	Reverse	TTTTCAGTCACTCACGTCGAGAC	
OsPT4-qRT	Forward	TTCTGCTAGTGTACCAAACAAAATTACA	
	Reverse	CTAAGTGGCATTTATAATATCAACAGTAACC	
OsMYB63-qRT	Forward	AGGACTTGGAGAAGGAGCT	
	Reverse	TTGGAAGTAGCACGACACTG	
OsACTIN-qRT	Forward	GAACTGGTATGGTCAAGGCTG	
	Reverse	ACACGGAGCTCGTTGTAGAAG	
OsMYB58-KO	P1	CGAACACGCAAGAATTAACT	Genotyping from RNAi-plants
	P2	GCTAAGCACACGTGTAGGAT	
	P3	GGTGAATGGCATCGTTTGAA	

Supplementary Table S2. The shoot and root biomass of Arabidopsis wild-type Col-0 and *OsMYB58*-AraOX plants

		Fresh weight of shoots (mg)	Fresh weight of roots (mg)	Length of primary roots (mm)	Number of lateral roots	Pi in shoots (nM/mg FW)	Pi in roots (nM/mg FW)
High Pi	Col-0	7.633±0.447	4.100±0.548	68.302±2.658	19.4±1.140	283.969±2.659	290.360±3.018
	OsMYB58-AraOX #1	6.567±2.702	3.100±1.140	57.964±4.080	16.4±1.140	270.724±5.673	199.466±5.064
	OsMYB58-AraOX #2	6.833±5.148	2.933±1.140	57.836±5.089	14.4±3.050	269.202±5.833	206.873±9.540
	OsMYB58-AraOX #4	6.067±0.548	2.900±0.894	52.584±3.651	13±0.707	247.971±6.081	196.415±1.983
	OsMYB58-AraOX #7	5.733±0.548	2.633±0.447	52.144±2.575	11.8±0.837	251.950±0.590	163.806±1.695
Low Pi	Col-0	3.267±0.548	2.067±0.548	49.102±0.643	16.4±2.408	217.457±1.494	249.371±1.944
	OsMYB58-AraOX #1	2.333±1.732	1.633±0.837	44.526±2.550	13.2±1.924	195.050±3.080	168.463±8.974
	OsMYB58-AraOX #2	2.400±1.949	1.500±1.000	43.496±2.453	12.8±3.033	202.268±5.161	174.302±9.552
	OsMYB58-AraOX #4	2.200±0.447	1.633±0.447	41.37±1.101	8.2±0.837	181.310±1.643	152.743±1.947
	OsMYB58-AraOX #7	1.800±0.447	1.333±0.707	41.096±1.180	8.8±0.837	151.431±0.589	140.571±3.936

Supplementary Table S3. The shoot and root biomass of rice wild-type, *OsMYB58*-OX, and *OsMYB58*-KO plants

		Fresh weight of shoots (mg)	Fresh weight of roots (mg)	Length of shoots (cm)	Length of primary roots (mm)	Length of seminal roots (cm)	Number of seminal roots	Length of lateral roots (cm)	Number of lateral roots	Length of root hair (mm)	Number of lateral roots per 1cm primary roots	Pi in shoots (nM/mg F.W.)	Pi in roots (nM/mg F.W.)
High Pi	WT	105.000 ±4.000	57.667 ±4.163	22.100 ±0.200	15.767 ±0.115	6.609 ±0.342	4.667 ±0.577	0.412 ±0.047	46.333 ±4.726	0.097 ±0.006	8.506 ±1.410	257.940 ±4.283	206.398 ±3.357
	OsMYB58-OX #5	54.333 ±2.082	37.000 ±2.000	14.867 ±0.252	8.533 ±2.023	2.428 ±0.452	4.000 ±1.000	0.140 ±0.028	12.667 ±2.517	0.042 ±0.013	2.878 ±0.656	184.824 ±1.089	148.252 ±0.864
	OsMYB58-OX #7	50.333 ±4.726	36.667 ±2.517	12.667 ±0.208	10.967 ±1.405	2.566 ±0.270	4.000 ±1.000	0.157 ±0.024	14.667 ±4.509	0.039 ±0.009	3.295 ±0.802	188.646 ±1.659	147.476 ±3.675
	OsMYB58-OX #13	64.333 ±3.512	41.333 ±1.528	17.223 ±0.611	14.000 ±1.217	2.786 ±0.150	3.667 ±0.577	0.216 ±0.031	13.667 ±3.055	0.051 ±0.017	4.226 ±0.975	191.851 ±2.762	155.583 ±7.174
	OsMYB58-KO #2	92.667 ±1.528	47.333 ±2.082	16.667 ±0.058	13.633 ±0.764	6.100 ±0.414	4.667 ±0.577	0.389 ±0.032	38.333 ±9.018	0.137 ±0.048	7.413 ±2.538	261.711 ±3.983	204.990 ±1.645
	OsMYB58-KO #6	93.667 ±2.517	48.667 ±2.082	16.367 ±0.306	13.567 ±0.404	6.146 ±0.773	4.333 ±0.577	0.394 ±0.059	39.333 ±12.503	0.125 ±0.010	7.227 ±2.411	255.733 ±1.399	204.243 ±1.339
	OsMYB58-KO #8	94.333 ±4.041	51.667 ±2.082	19.700 ±0.173	15.700 ±1.044	6.764 ±0.262	4.333 ±0.577	0.408 ±0.033	41.333 ±11.590	0.139 ±0.033	8.530 ±0.794	261.241 ±1.966	205.740 ±5.046
Low Pi	WT	86.000 ±2.646	47.000 ±4.583	19.900 ±0.200	14.133 ±0.321	5.151 ±0.095	5.667 ±1.155	1.183 ±0.044	154.333 ±3.055	0.352 ±0.037	26.724 ±0.464	190.506 ±3.224	131.529 ±0.673
	OsMYB58-OX #5	50.667 ±0.577	35.333 ±2.517	13.400 ±0.265	6.633 ±1.012	3.477 ±0.565	3.333 ±0.577	0.510 ±0.087	72.333 ±9.074	0.149 ±0.014	17.849 ±2.261	133.793 ±2.267	88.158 ±2.251
	OsMYB58-OX #7	42.667 ±2.082	36.667 ±1.528	12.300 ±0.794	8.467 ±0.945	3.177 ±0.200	3.667 ±0.577	0.551 ±0.063	74.333 ±6.658	0.156 ±0.026	18.135 ±1.846	140.147 ±1.974	91.022 ±1.770
	OsMYB58-OX #13	59.333 ±2.517	40.333 ±3.055	14.733 ±0.551	10.400 ±0.917	3.043 ±0.115	3.667 ±0.577	0.650 ±0.068	76.667 ±7.506	0.168 ±0.032	17.647 ±3.093	138.850 ±2.137	94.746 ±0.719
	OsMYB58-KO #2	73.333 ±3.512	48.333 ±1.528	15.267 ±0.115	14.500 ±0.265	6.005 ±0.170	6.333 ±0.577	1.013 ±0.162	178.000 ±9.644	0.555 ±0.076	34.011 ±0.615	190.729 ±0.490	134.402 ±0.476
	OsMYB58-KO #6	72.667 ±2.082	48.667 ±2.517	15.800 ±0.100	14.333 ±0.777	6.679 ±0.209	6.667 ±1.528	0.995 ±0.096	179.333 ±7.371	0.545 ±0.057	31.644 ±2.706	193.204 ±1.512	132.225 ±2.793
	OsMYB58-KO #8	72.667 ±2.517	48.333 ±2.517	16.233 ±0.651	15.833 ±1.102	6.577 ±0.238	6.333 ±1.155	1.080 ±0.179	182.333 ±10.786	0.573 ±0.083	31.021 ±2.767	185.723 ±2.847	131.000 ±1.125
